# Natural Compounds Affecting Inflammatory Pathways of Osteoarthritis

**DOI:** 10.3390/antiox11091722

**Published:** 2022-08-30

**Authors:** Yi Ting Lee, Mohd Heikal Mohd Yunus, Azizah Ugusman, Muhammad Dain Yazid

**Affiliations:** 1 Department of Physiology, Universiti Kebangsaan Malaysia, Cheras, Kuala Lumpur 56000, Malaysia; 2 Centre for Tissue Engineering Regenerative Medicine, Universiti Kebangsaan Malaysia, Cheras, Kuala Lumpur 56000, Malaysia

**Keywords:** natural compounds, osteoarthritis, ROS, oxidative stress, antioxidants, anabolic/catabolic effects

## Abstract

Osteoarthritis (OA) is the most common type of arthritis and chronic joint disease, affecting more than 240 million people worldwide. Although there are numerous advances in using drugs in treating OA, the use of natural compounds has aroused much interest among researchers due to their safety margin. Recent discovery shows that natural compounds play an extensive role in the oxidative stress signaling pathway in treating OA. Thus, this review summarizes the commonly used natural compounds for treating OA focusing on the oxidative stress signaling pathway and its downstream mediators. Selected databases—such as Scopus, Web of Science, Nature, and PubMed—were used to search for potentially relevant articles. The search is limited to the last 15 years and the search was completed using the Boolean operator’s guideline using the keywords of natural product AND oxidative stress AND osteoarthritis OR natural extract AND ROS AND degenerative arthritis OR natural plant AND free radicals AND degenerative joint disease. In total, 37 articles were selected for further review. Different downstream mechanisms of oxidative stress involved in the usage of natural compounds for OA treatment and anabolic and catabolic effects of natural compounds that exhibit chondroprotective effects have been discussed with the evidence of in vitro and in vivo trials in this review.

## 1. Introduction

Osteoarthritis (OA) is the most common type of arthritis that affects joints and is one of the chief sources of disability [1]. As claimed by Katz et al. (2021), it is estimated that OA affects more than 240 million people globally and up to 32 million in the United States alone [2]. According to Musumeci et al. (2015), OA affects roughly half of the population over 65 years old with a more noteworthy rate in females after menopause (18%) compared to males (9.6%) [3]. This draws public concern due to its negative impact on irreversible physical disability [4]. The progression of OA is usually slow and persistent for a longer period [5]. OA usually damages the joints and their surrounding tissues, which play a crucial role in the movement. This includes bones, meniscus, tendons, ligaments, synovium, and articular cartilage. OA can affect any joint in the body but predominantly affects the knees, hands, feet, and hips [2]. Permanent articular cartilage degradation, osteophyte formation, inflammation of synovium with various degrees, remodeling of subchondral bone, ligament and meniscus degeneration, cartilage calcification, angiogenesis and joint capsule hypertrophy [6,7,8], changes in local fat pads, nerves, periarticular muscles, and the bursa [6] are some of the pathological changes that are commonly notable in OA affected joints.

OA can be classified into two types, which are primary and secondary OA. Primary OA—also known as idiopathic OA—refers to the deteriorating changes of joints that occur due to genetic conditions but without known underlying causes. The primary OA can be further classified into localized OA, which affects only one joint while the generalized OA affects three or more joints. Secondary OA is associated with risk factors or injuries. Some of the relatable condition of secondary OA includes obesity, diet, diabetes, physical activities, rheumatoid arthritis, and other diseases related to bone or metabolism [3,9].

Hitherto, there is no effective cure to stop or reverse the progression of OA. The current goals for OA management are mainly to alleviate painful symptoms, reduce disability, and improve the overall quality of life. Some of the non-pharmacological management methods for OA include weight management, exercise, and strength training [10,11,12]. The treatment for OA is always a lifelong therapy. However, long-term usage of OA-relieving medicines could cause negative effects on various organs specifically in the kidney, gastrointestinal tract, and cardiovascular system [13,14]. Thus, there is an immediate demand in clinical practice for an OA symptom-relieving treatment strategy that is desirable for long-term use with minimum adverse effects.

In consideration of the side effects of OA medicines, the possibility of natural compounds that are able to exhibit chondroprotective effects in the oxidative stress signaling pathway has gained researchers’ attention. This is because these natural compounds have been proven to act as osteoprotective and chondroprotective capabilities by acting as antioxidants and anti-inflammatory agents, preventing chondrocyte catabolism, and suppressing chondrocyte differentiation, thus improving bone health [5,15]. Besides that, natural compounds are considered a safer alternative for OA treatment due to their lesser or non-existent side effects [16,17]. Therefore, this review recapitulated the chondroprotective effects of the natural compound on OA from the oxidative stress signaling pathway perspective.

## 2. Materials and Methods

### 2.1. Search Strategy

**Figure 1:**
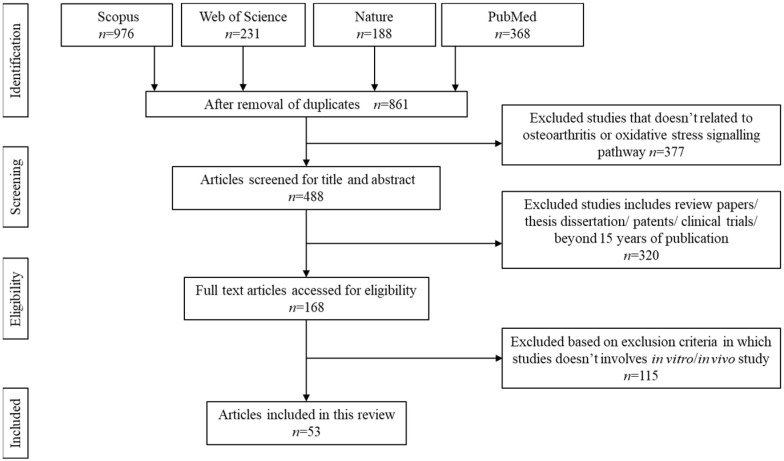
Article search and selection process.

The search was carried out systematically using Preferred Reporting Items for Systematic Reviews and Meta-Analyses (PRISMA) guideline for scoping review as described elsewhere. Boolean operator guideline was used to select the appropriate keywords for article search. The selected keywords are: natural product AND oxidative stress AND osteoarthritis OR natural extract AND ROS AND degenerative arthritis OR natural plant AND free radicals AND degenerative joint disease. The articles were searched using Nature, PubMed, Scopus, and Web of Science databases. Figure 1 summarises the search strategy.

### 2.2. Inclusion Criteria

All original articles that are based on in vitro or in vivo studies were chosen to be further reviewed. Only articles written in the English language were selected. The article selections were limited to the last 15 years starting from 2007 onwards to obtain the latest published research papers. The articles must contain either four of the following parameters: (1) subject, (2) active compounds, (3) type of model used, (4) dosage, (5) duration of treatment, and (6) outcome observed.

### 2.3. Exclusion Criteria 

Articles that did not meet the inclusion criteria, theses, secondary articles, review papers, clinical trials, conference proceedings, and patents were excluded from being further reviewed. In addition, any articles that do not provide clear information on dosage or duration of treatment were excluded. Data extraction was carried out independently by three authors (L.Y.T., A.U., and M.D.Y.) and any disagreements were resolved by consulting the fourth author (M.H.M.Y.). No conflict of interest arises among the authors during the discussion or the data extraction process.

## 3. Results

The initial search resulted in 1763 articles. Upon screening, 861 articles were removed due to duplication, unmatched content, or absence of an abstract. Another 377 articles were further removed due to unreliable keywords or articles discussing stiffness or knee injuries apart from osteoarthritis. Additionally, 320 articles were removed upon confirming these papers as review papers, technical papers, and retrospective studies. A total of 53 articles that met the inclusion criteria were chosen to be further reviewed. 

## 4. Pathogenesis of OA

**Figure 2:**
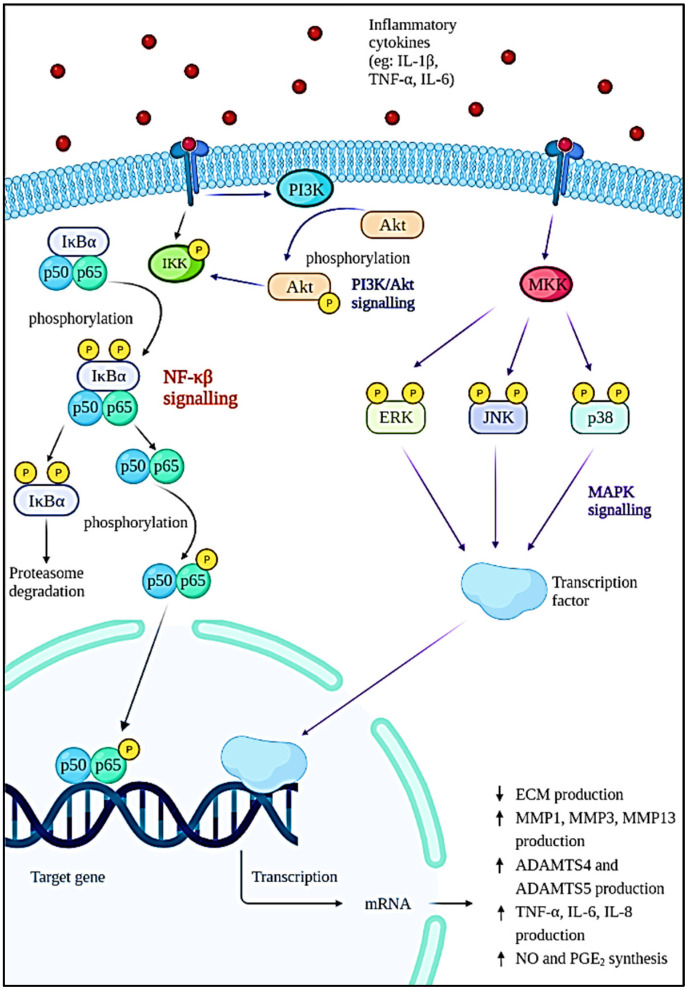
Intracellular signaling pathways and downstream effects involved in the progression of OA.

The pathophysiology of OA is complex and poorly understood. Generally, normal articular cartilage consists of chondrocytes and extracellular matrices (ECM) such as collagen, proteoglycans, and water to provide the shock-absorbing capability [18,19]. Proteoglycan consists of aggrecan and hyaluronan that interact with each other to form glycosaminoglycan (GAG) [20]. The failure of chondrocytes to establish equilibrium between ECM synthesis and degradation will result in osteoarthritis. In these cases, the wear particles produced in joints will be phagocytosed by the immune cells, resulting in the increased secretion and activation of lysozymes during injury [21]. When the wear particles’ formation outpaces the system’s ability to remove them, they will mediate the inflammation process, thus causing the chondrocytes to produce degradative enzymes to break down collagen and proteoglycan in the joint [22]. As a natural defence of immune response, various immune and pro-inflammatory cells—including neutrophils, megakaryocytes, macrophages, lymphocytes, leukocytes, and dendritic cells—will be produced [23]. A network of factors—including proinflammatory cytokines, chemokines, and lipid mediators—is responsible for bind to chondrocytes and coordinating the immune cell function by activating signal transduction pathways (Figure 2), especially the nuclear factor kappa-light-chain-enhancer of activated B cells (NF-κβ) pathway [24]. As a result, more metalloproteinases will be formed while type II collagen production is inhibited. Resultantly, degradation of cartilage is accelerated, thus increasing chondrocyte apoptosis [18]. On the contrary, various cytokines and chemokines play an important role in the pathogenesis of OA. This includes inflammatory cytokines such as interleukin (IL)-1β (IL-1β), tumour necrosis factor-alpha (TNF-α), IL-6, IL-15, IL-17, and IL-18 [25,26]. Among them, IL-1β, TNF-α, and IL-6 are the most significant inflammatory mediators in the etiology of OA, as they trigger a variety of signaling pathways, which in turn activate other cytokines and pathogenic processes. Apart from that, cytokines are also able to stimulate chemokine production and this will attract more inflammatory cells to the joint, thereby further enhancing the secretion of inflammatory substances and accelerating the progression of the disease [27].

The NF-κβ transcription factor is a flexible and multifunctional transcription factor that is involved in immunological responses, cellular differentiation, inflammatory responses, and also normal and malignant cell survival [28]. It has a role in a variety of biological activities. In OA, the NF-κβ transcription factor is abnormally activated and it is known to be one of the disease-causing factors. This factor is initiated by various inflammatory cytokines such as IL-1β, TNF-α, and IL-6; severe mechanical stressors; or the breakdown products of ECM that can promote the transcription of the catabolic gene and also stimulate the production of inflammatory mediators through a positive feedback loop [29,30]. The activation of IκB kinase (IKK) leads to IκBα phosphorylation and degradation by the proteasome, which is the first step in the NF-κβ signaling cascade. The p65 protein is liberated from the cytoplasm, phosphorylated, and translocated to the nucleus. The genes of degradative enzymes and aggrecanases, a disintegrin and metalloproteinase domain with thromboplastin motifs’ (ADAMTS), including matrix metalloproteinases (MMP)-1, MMP3, MMP-13, ADAMTS4, and ADAMTS5 are activated as a result of these processes. This will eventually lead to the breakdown of collagen and aggrecan [31,32]. The expression of cyclooxygenase 2 (COX-2) and inducible nitric oxide synthase (iNOS), which are the major proinflammatory and destructive mediators of OA, will also be stimulated. The upregulation of iNOS and COX-2 increased nitric oxide (NO) and prostaglandin E2 (PGE_2_) production [29,33,34]. The increase in NO will modulate the expression of cytokines and homeostasis of ECM by inhibiting the production of proteoglycan and collagen type II, which will result in oxidative damage and chondrocyte death. The elevation of PGE_2_ lowers the synthesis of ECM, inhibits the proliferation of chondrocytes, and leads to osteophyte formation [26,35,36].

According to Xie et al. (2019), the phosphoinositide 3-kinase (PI3K)/protein kinase B (Akt) signaling is known to have a synergistic effect on the NF-κβ signaling pathway. This pathway is activated when cytokines such as IL-1β bind to their receptors on the cell surface. The phosphorylation of Akt by membrane protein PI3K will occur in response to the stimulation and cause the cell to synthesize more MMPs [37]. In addition, the mitogen-activated protein kinase (MAPK) pathway is one of the multiple signal transduction pathways in the OA progression [38]. The three signal cascades pathway of the MAPK pathway includes p38, extracellular signal-regulated kinase (ERK), and c-Jun N-terminal kinase (JNK) [39]. These are the mediator that controls the downstream MMPs and pro-inflammatory cytokines expression. Cytokines, matrix proteins, and growth factors can activate MAP kinases (MKKs) by binding to integrin, cytokine receptors, G-protein coupled receptors, and receptor tyrosine kinases. Upon binding to the cell surface receptors, signals known as ‘upstream activators’ are generated and will cause phosphorylation of specific MKKs by MAP kinase. The activated MKKs will then trigger the activation of transcriptional regulatory proteins resulting in the increased expression of inflammatory genes such as TNF-α, IL-1, and MMPs. The cytokines produced can then keep JNK activated, eventually resulting in increased production of MMPs and cytokines [40].

### Inflammatory Pathway in Osteoarthritis Pathogenesis

The progression of OA mainly depends on the reactive oxygen species (ROS) and oxidative stress. ROS production is one of the essential steps for the regulation of homeostasis in the chondrocytes. ROS is produced in the cells via NADPH oxidase and mitochondria in the chondrocytes. ROS usually modulates the normal expression of genes, apoptosis, synthesis of ECM, and production of inflammatory factors such as cytokines. The overexpression of NADPH oxidase enhances the build-up of oxidative stress in the joints, thereby accelerating the progression of OA [41]. 

Besides that, the upregulation of inflammatory cytokines—such as IL-1β, IL-17, interferon-γ (IFN-γ), and TNF-α—via iNOS further worsens the OA condition. Protein synthesis of iNOS is known as one of the nitrogen metabolites, the key enzyme for NO formation, and a primary mediator of inflammation in skeletal muscles [42]. Overexpression of iNOS will stimulate abnormally high production of NO in cartilages which then will cause damage to the cartilage due to its role as an endogenous modulator in cartilage matrix turnover. Thus, the cartilage tends to lose chondrocyte phenotypes through the dedifferentiation of fibroblastoid [43]. Aside from this, NO together with IL-1 could inhibit proliferation in the chondrocytes and stimulate apoptosis. This is mainly because IL-1 increases the vulnerability of NO toward oxygen radical-mediated cell apoptosis in chondrocytes [44]. Therewithal, NO suppresses the synthesis of proteoglycan by preventing the attachment of chondrocytes to fibronectin in the ECM. This will compromise the cell survival, hence causing chondrocyte apoptosis [45]. 

**Figure 3:**
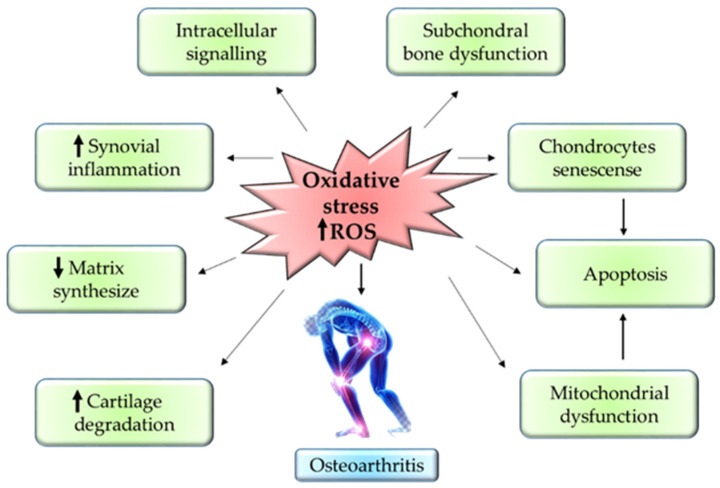
Mechanism of oxidative stress in OA pathogenesis.

Into the bargain, the formation of hydrogen peroxide (H_2_O_2_) by hypoxanthine-xanthine oxidase induces apoptosis in chondrocytes through ROS in a dose-dependent manner. In such cases, the membrane integrity of the mitochondria is compromised, thus causing the release of cytochome c into the cytoplasm [46]. The elevation of cytochome c in the cytoplasm will eventually stimulate the activation of apoptotic protease-activating factor-1. This will then configure the apoptosome through oligomerisation with Apaf-1, thus initiating caspase-9 [47]. Stimulation of caspase-9 will cleave caspase-3, also known as ‘executioner caspase’, leading to chondrocyte apoptosis [48]. Conversely, sodium nitroprusside (SNP), a donor of NO, triggers an apoptotic mechanism by suppressing the action of BCL-2. SNP induces apoptotic mechanisms in chondrocytes by stimulating DNA fragmentation, dysfunctional mitochondria, and remodeling of the cytoskeleton [41]. Figure 3 summarizes the inflammatory pathway related to pathogenesis in OA. Figure 3 summarizes the inflammatory pathway related pathogenesis in OA. 

## 5. In Vitro and In Vivo Evidence of Natural Compounds Affecting Inflammatory Pathways of Osteoarthritis

The findings from this review show that 100% of the natural compounds can slow down the progression of OA by targeting the oxidative stress signaling pathway as shown in Table 1 and Table 2. Out of 37 studies, 32 studies were conducted in vitro only or in vitro followed by in vivo study with a 100% success rate in cartilage regeneration or inhibition of the chondrocytes damage. This involves various pathways under the oxidative stress signaling mechanism. The most common discussed pathway in the in vitro studies includes inhibition of p65, AKT, MAPK (ERK1/2 and p38), apoptosis signal-regulating kinase 1 (ASK1), JNK, TNF, NF-κβ, and IκBα phosphorylation. Generally, ROS—being a second messenger—is able to activate ROS/MAPK, ROS/AKT, and TNF/ NF-κβ in OA pathogenesis [49]. In vitro study proves that natural compounds such as galangin [50,51], propolis [52], astaxanthin [53], anthriscus sylvestris [54], cynaroside [55], and rhizoma coptidis [56] are able to suppress the production of ROS and its downstream pathway to prevent the pathological progression of OA. 

Conversely, cytoprotective genes become stimulated due to the upregulation of nuclear factor (erythroid-derived 2)-like 2 (Nrf-2) in proper regulation of oxidative stress [57]. Nrf-2 plays a vital role in the regulation of redox homeostasis via the modulation of the antioxidant response element (ARE). In such conditions—under excessive production of ROS—Nrf-2 will be downregulated leading to the depletion of SOD, GPx, and GSH. However, in a controlled oxidative stress environment, Nrf2 exhibits cytoprotective activity thus causing the chondrocytes to remain ‘young’ [58]. Another study supports the statements by indicating that chondrocytes treated with astaxanthin show an upregulation of Nrf2, which in turn increases the expression of heme oxygenase-1 (HO-1) and reduces the activity of NADPH and nicotinamide adenine dinucleotide phosphate (NADPH):quinone oxidoreductase (NQO1) [59]. The stimulation of HO-1 will in turn repress the expression of the MMP gene in the chondrocytes [60]. Similarly, a reduction in NADPH is able to enhance proteoglycan synthesis in the cartilage [61]. NQO1 act as detoxifying enzyme or defence mechanisms for unregulated antioxidants [62]. Aside from this, in vitro analysis of chondrocytes treated with curcumin [63] and EGCG [64] shows gradual elevation of CITED 2 gene. This proves that natural extracts such as curcumin and EGCG are able to act as chondroprotectants by suppressing the activity of MMP through the expression of CITED 2 gene via the JAK/STAT pathway. CITED 2 gene is also known as a transcriptional regulator that plays an important role in the downregulation of MMPs [65]. 

**Figure 4:**
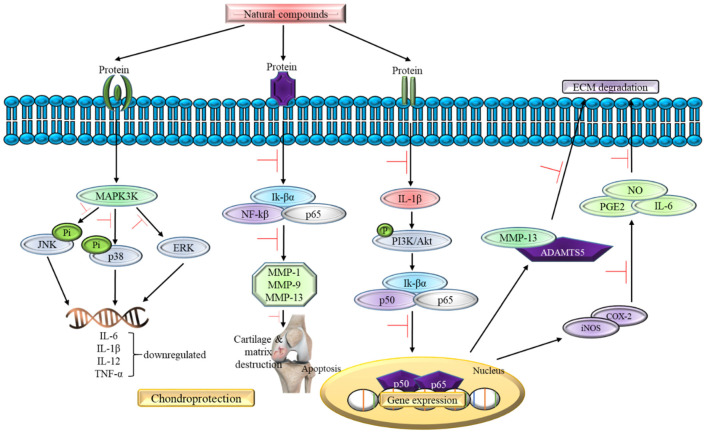
The role of natural compounds in the inflammatory pathway of OA.

In addition, 20 studies reviewed in this article focus on the in vivo study as shown in Table 2 using the osteoarthritis-induced rat model. The data analysis shows no contraindication observed in the natural compounds-exposed rats and only positive outcome has been documented so far. For instance, all 20 studies supported the claim that natural compounds enhance chondroprotective action regardless of different downstream pathways of oxidative stress signaling pathway involvement. For instance, oral administration of natural compounds (galangin and propolis) is able to reverse articular degradation as early as 14 days [51,52], while a reduction in MMP-2 activity is recorded within 5 h of intraperitoneal injection of *Cryptolepis buchanani* [66]. Five studies show a reduction in ROS, lipid peroxidation, and inflammatory factors such as IL-1β, IL-6 and TNF-α, which in turn mitigates OA [51,52,54,67,68]. One in vivo study states that there is a gradual decrease in GAG and hydroxyproline levels in chondrocytes treated with natural compounds [52]. In the clinical setting, GAG and hydroxyproline could reflect the range of cartilage damage and a reduced level indicates a positive outcome in the treated rats. In summary, all in vivo studies show prevention in the articular degradation at the end phase of their study, thus supporting the data obtained in the in vitro experiments. Figure 4 summarizes the effect of natural compounds on the inflammatory pathway in OA.

### Role of Natural Compounds in Oxidative Stress Signaling Pathway in Osteoarthritis 

Natural compounds either from plant extracts or active compounds, bring unlimited prospects for the development of new therapeutic agents for OA due to difference in phytochemical variety. Table 1 and Table 2 summarize the chondroprotective effects of various natural compounds.

## 6. Role of Natural Compounds on Oxidative Stress Signaling in OA

### 6.1. Aconitum carmichaelii Debx

*Aconitum carmichaelii* Debx. belongs to *Aconitum* genus and Ranunculaceae family. Europe, North America, and Asia are among the temperate zones of the northern hemisphere where they can be found [109]. Since ancient times, the ‘Chuan Wu’, the mother roots of *A. carmichaelii* is used to treat rheumatism and pains [110]. The effect of the oxidative stress signaling of *A. carmichaelii* on OA has been proven through several in vitro and in vivo studies. In accordance with the study by Tong et al. (2014) [81] on intra-articular mono-iodoacetate (MIA)-induced OA rat model and MIA-treated rat chondrocytes, the detoxicated ‘Fuzi’ is able to promote the proliferation of chondrocytes and suppress chondrocytes damage by MIA in vitro and show chondroprotective activity in vivo by preventing abnormalities in the subchondral bone, thus preserving the morphology of joint and density of the bone [81,111]. 

This result is also supported by Zhang et al. (2020) [67] who carry out the study by using processed *A. carmichaelii* Debx. lateral root with peel or also known as ‘Hei-Shun-Pian’ (Hsp). This detoxified Hsp (dHsp) can significantly reduce joint pain and also protect the articular cartilage from degeneration. According to the result of an in vivo experiment, dHsp can restore the MIA-induced upregulation of collagen type X, MMP2, SOX-5, ADAMTS4, ADAMTS5, and ADAMTS9 genes expression. A similar result was shown in an in vitro experiment using TNF-α-treated rat articular chondrocytes by increasing the aggrecan messenger ribonucleic acid (mRNA) level and reducing the MMP1 and ADAMTS9 mRNA expressions. This study also indicates that the benzoylaconitine and benzoylhypaconite in dHsp play roles in this chondroprotective effect [67]. Collectively, these results suggest that the extract of *A. carmichaelii* may be a potential antioxidant agent for treating OA.

### 6.2. Anthriscus sylvestris (L.) Hoffm

*Anthriscus sylvestris* (L.) Hoffm, also known as cow parsley or wild chervil, is from the family of the Apiaceae (Umbelliferae). It is widely distributed around the world, such as roadsides and shrubbery, but mainly in northern temperate zones and at high altitudes in the tropical jungle [112]. This plant is proven to play a vital role in oxidative stress signaling in OA in various studies. For instance, a positive outcome is seen on IL-1β-treated rat chondrocytes and destabilization of the medial meniscus (DMM) surgery-induced OA rat model. The leaves extract from *A. sylvestris* has shown the ability to repress nitrite, PGE_2_, iNOS, and COX-2 genes expression and also the expression and protein level of matrix-degrading enzymes (MMP3, MMP13, ADAMTS4) induced by IL-1β. The chondrocytes treated with plant extract also prevent collagen type II, aggrecan, and proteoglycan degradation. The IκBα, ERK, JNK, and p38 phosphorylation is also being suppressed by the *A. sylvestris* aqueous extract and causes inactivation of NF-κβ and MAPK signaling pathways [113]. 

Moreover, the rat model treated with the plant extract showed lesser cartilage destruction and proteoglycan degradation compared to the OA group [54]. Cynaroside has been identified as one of the key chemicals that possess anti-inflammatory in the extract of *A. sylvestris* and is believed to play a major role in the chondroprotective effect of *A. sylvestris*. This statement is supported by another study by Lee et al. in 2020. Cynaroside is able to suppress the expression of catabolic factors including nitrite, iNOS, ROS, PGE_2_, COX-2, MMP1, MMP3, MMP13, and ADAMTS4, as well as prevent aggrecan and collagen type II degradation. In addition, it can also inhibit MAPK and NF-κβ pathways, which play a role during inflammation. The ex vivo results revealed that the production of nitrite, PGE_2_, iNOS, COX-2, ADAMTS4, and MMP13 can be inhibited by cynaroside, which is consistent with the results obtained via an in vitro study. Besides that, it can also protect the proteoglycan from degradation [55,95]. The results obtained provide a promising candidate for the alleviation of OA focusing on the oxidative stress signaling pathway.

### 6.3. Artemisia argyi H. Lév. and Vaniot

*Artemisia argyi (A. argyi)* is a creeping rhizome herbaceous perennial plant from *Artemisia* L. genus, Asteraceae family. This plant is known as ‘aiye’ in China and ‘gaiyou’ in Japan. It is indigenous to China, Japan, and the former Soviet Union’s far eastern regions. *A. argyi* has been shown to have antioxidant and anti-inflammatory characteristics [114]. The role of chondroprotective of *A. argyi* in oxidative stress signaling was proven through an in vitro, ex vivo, and in vivo experiment by Lee et al. (2020) using seomae mugwort, *A. argyi* from Korean native. The in vitro experiment revealed that the extract is able to suppress the MMP3, MMP13, ADAMTS4, and ADAMTS5 expressions and also reduce the activity of aggrecanase induced by IL-1β, IL-6, and TNF-α. The extract of seomae mugwort also showed its ability in preventing the degradation of sulfate proteoglycan in IL-1β-treated cartilage explants. The extract suppresses the destruction of cartilage and reduces the thickness of subchondral bone in the in vivo experiment of the DMM-induced OA model [95].

The results from high-performance liquid chromatography (HPLC) reported that the extract of *A. argyi* consists high amount of jaceoside and this compound is able to reduce the expression of MMP3, MMP13, ADAMTS4, and ADAMTS5. Both the extract and jaceosidin are able to block the degradation of IκBα and NF-κβ activation. However, the suppression of JNK phosphorylation was only seen in the experiments which used seomae mugwort extract. These findings indicate the chondroprotective properties of the *A. argyi* extract and jaceosidin, which is the active component, as a potential candidate for managing OA [95].

### 6.4. Edible Bird’s Nest (EBN)

Edible bird’s nest is a dried viscous discharge of male swiftlets from several swiftlet species’ salivary glands including the *Aerodramus* and *Collocalia* (Apodidae) genus during the breeding season [115]. Chua et al. (2013) have demonstrated the chondroprotective properties of the extract of EBN by targeting oxidative stress in OA. This extract was able to suppress the expression of catabolic genes of MMP1, MMP3, IL-1, IL-6, IL-8, COX-2, and iNOS. The chondrocytes when cultured with EBN extract also showed a significant reduction in PGE_2_. The gene expression of collagen type II, aggrecan, and SOX-9 increased in the chondrocytes culture supplemented with EBN extract. The production of sulfated glycosaminoglycan (sGAG) was also elevated in the assessment of anabolic activity [79]. The result concluded that the extract of EBN possesses antioxidant characteristics and can be a potential agent for OA treatment. 

### 6.5. Stichopus chloronotus

The *Stichopus chloronotus (S. chloronotus)* aqueous extract (SCAE) has been proven to show a chondroprotective effect on OA articular chondrocytes by targeting the oxidative stress signaling pathway. Thus, SCAE increased the expression of collagen type II, aggrecan, and SOX-9, which is the marker specific for cartilage. Moreover, it successfully suppresses the expression of IL-1, IL-6, IL-8, MMP1, MMP3, MMP13, COX-2, iNOS, proteinase-activated receptor 2 (PAR-2), and collagen type I. This extract is also able to reduce the PGE_2_ and NO production and increases the production of sGAG. This result hence suggests that SCAE is a good anti-oxidative, anti-inflammatory, and pro-chondrogenic agent [93]. Incongruent with Mou et al. (2018) [116], *S. chloronotus* consists of fucosylated chondroitin sulfate (fCS), a polysaccharide with high sulfate content, that has numerous biological properties including anti-inflammatory and antioxidant properties [116,117]. Chondroitin sulfate is a natural glycosaminoglycan, which is an essential proteoglycan component found abundantly in the cartilage and ECM [118]. The use of fCS on OA patients resulted in slow OA progression, increase cartilage regeneration, and anti-inflammatory activity [119,120,121]. This further supports *S. chloronotus* as a potential antioxidant agent to treat OA.

### 6.6. Galangin

Galangin, also known as norizalpinin, is a 3,5,7-trihydroxyflavone with a molecular formula of C_15_H_10_O_5_ and a molecular weight of 270.24 g/mol. It can be found abundantly in propolis, honey, the root of *Alpinia officinarum*, and the aerial parts of *Helichrysum aureonitens* [122]. The chondroprotective ability of galangin in oxidative stress signaling mechanism has been demonstrated by several studies. In the study by Huang et al. (2021), the in vitro experiment results showed that galangin can reduce the catabolic factors and inflammatory cytokines expression including iNOS, COX-2, MMP1, MMP3, MMP13, and ADAMTS5 and also prevent aggrecan and collagen type II degradation induced by IL-1β. Furthermore, galangin also restricts the Akt, IKKα/β, IκBα, and p65 phosphorylation that play roles in the activation of Akt and NF-κβ signaling. In the anterior cruciate ligament transection (ACLT) rat model, injection of galangin intra-articulary can prevent deterioration of cartilage [50]. 

Su et al. (2021) supported the therapeutic effect of galangin on OA using an MIA-induced OA rat model. The rat model receiving galangin supplementation showed a significantly reduced level of urinary collagen type II, lipid peroxidation, ROS, IL-1β, IL-6, and TNF-α. Additionally, the level of catalase, superoxide dismutase (SOD), glutathione peroxidase (GPx), and reduced glutathione (GSH) is being upregulated showing that galangin possesses an antioxidant effect. Galangin also inhibits cartilage breakdown as the mRNA and protein expression of collagen type II are reduced after treatment [51]. 

The use of galangin as an antioxidant agent is also being supported by a study by El-Ghazaly et al. (2011) using aqueous extract of propolis (AEP) as galangin is the major component in propolis. The use of AEP as the treatment in Freud’s adjuvant injected rat model showed reduced cartilage degradation. The reduction of sGAG and hydroxyproline level is being prevented by AEP and the cartilage oligomeric matrix protein (COMP) further support these results. In addition, the level of TNF-α is being reduced and the level of NO and oxidative stress biomarkers such as GSH and malondialdehyde (MDA) is maintained by the AEP [52]. All results obtained above proved that galangin can be a promising novel antioxidant agent in treating OA.

### 6.7. Astaxanthin

Astaxanthin (3,3′-dihydroxy-β,β’-carotene-4,4′-dione), a red xanthophyll carotenoid with a molecular weight of 596.8 g/mol, is a secondary metabolite that gives a variety of marine animals and microorganisms their red-orange hue. Astaxanthin showed its chondroprotective properties in several in vitro and in vivo experiments by targeting the oxidative stress signaling pathway. For instance, in an in vitro experiment by Chen et al. (2013) on IL-1β-treated human chondrocytes, astaxanthin inhibits MMP1, MMP3, and MMP13 expressions. It also blocks p38 and ERK1/2 phosphorylation and the degradation of Iκβα that are responsible for the activation of MAPK and NF-κβ signaling pathways [53]. Sun et al. (2019) also suggest that astaxanthin can protect against OA. In the in vitro study, astaxanthin reverses the changes caused by IL-1β, TNF-α, and tert-butyl hydroperoxide (TBHP) by reducing the level of MMP3, MMP13, and ADAMTS5 [59]. 

Furthermore, a study by Sun et al. (2019) [59] proves that this carotenoid is able to upregulate the Nrf-2 signaling and the downstream chondroprotective genes including HO-1 and reduced NQO1. The results also showed that astaxanthin reduced the level of iNOS and COX-2 protein upregulation by IL-1β and inhibit MAPK signaling by blocking the ERK and JNK phosphorylation. Furthermore, it also minimized the degradation of ECM and apoptosis stimulated by TNF-α by preventing activation of NF-κβ signaling through inhibiting phosphorylation of p65 and IκBα. The chondrocytes treated with astaxanthin also showed downregulation of TBHP-induced increase in ROS production that causes cell apoptosis. The apoptosis has been prevented as the increase in B-cell lymphoma 2 (Bcl-2)-associated X protein (BAX) and cleaved-caspase 3 and decrease in Bcl-2 protein level caused by OA inducing agents have been reversed. An in vivo model showed that astaxanthin successfully attenuated the breakdown of cartilage by showing less proteoglycan loss and cartilage erosion. These suggested that astaxanthin could protect the cartilage against OA, thus implying that it could be a promising therapeutic antioxidant agent for OA [59]. 

### 6.8. Berberine 

Berberine (BBR) is a quaternary ammonium salt, with a molecular weight of 336.36 g/mol and a molecular formula of C_20_H_18_NO_4_, produced from isoquinoline alkaloid. The chondroprotective effects of BBR in the oxidative stress signaling pathway have been proven in several studies through in vitro and in vivo experiments. In the in vitro experiment using connective tissue growth factor (CNN2)-induced IL-1β expression treated human synovial fibroblast, the BBR reduces the expression of IL-1β that play a role in inflammation. Moreover, the generation of ROS is reduced, as well as the phosphorylation of apoptosis signal-regulating kinase 1 (ASK1), p38, JNK, and p65 [56]. While in another in vitro test, the sGAG release and NO production are inhibited by BBR in IL-1β-treated rat articular chondrocytes and cartilage explant. In addition, the MMP1, MMP3, and MMP13 productions are being suppressed by BBR together with the increase in tissue inhibitors of metalloproteinases-1 (TIMP-1). A decrease in the severity of cartilage lesions is also being observed in the BBR treatment group [75]. 

Liu et al. (2015) also showed that BBR is able to inhibit the progression of OA in a collagenase-induced OA (CIOA) rat model. The rat treated with BBR shows a significant reduction in swelling, width, and stiffness of the knee and BBR also lowered the level of IL-1β in the serum. Moreover, the Safranin O staining showed that BBR can help preserve the proteoglycan compared with the control group [56]. For the in vivo work by Hu et al. (2011), the chondroprotective effect of BBR in the oxidative stress metabolites is in a dose-dependent manner. The expression of MMP1, MMP3, and MMP13 is being reduced and the TIMP-1 mRNA level is being upregulated in the BBR treatment group [75]. These results suggest that BBR may become one of the novel therapeutic strategies for OA management by focusing on the antioxidant mechanism.

### 6.9. Sesamin

The seed of sesame (*Sesamum indicum* L.) is one of the traditional health foods that is being widely utilized in East Asia a long time ago, but its role in OA is just recently being explored. As stated in the study by Phitak et al. (2012), the cartilage explants treated with sesamin showed reduced release of sGAG, hydroxyproline, and uronic acid (UA) indicating that sesamin can prevent the breakdown of proteoglycan. In addition, the expression and activity of MMP1, MMP3, and MMP13 are downregulated while p38 and JNK signaling pathways are being inhibited [78]. Similar results were found in a study by Kong et al. (2016) using human chondrocytes; however, this study focuses on the NF-κβ signaling pathway. The activation of the NF-κβ signaling pathway is being blocked as the sesamin inhibits the phosphorylation of p65 and IκBα, which play an important role in NF-κβ signaling. Furthermore, the sesamin showed its ability in protecting chondrocytes by enhancing the Nrf-2 and HO-1 expressions [87]. In the OA rat model, sesamin is able to reverse the pathological changes induced by papain. It reduces the disorganisation of cartilage and increases the thickness of cartilage. Aside from that, sesamin not only prevents the loss of collagen type II and proteoglycan but also increases their formation [78]. 

### 6.10. Wogonin

Wogonin (5,7-dihydroxy-8-methoxyflavone) is a natural flavonoid derived from the root and entire *Scutellaria baicalensis*, the leaves of *Andrographis paniculata* (Burm.f.) Nees, and stems of *Anodendron affine* (Hook. Arn.) Druce. It is well known for its antioxidant and anti-inflammation properties, particularly in metabolites of ROS [90,123,124]. This was proven through in vitro and in vivo studies by Khan et al. (2017) [90,123]. The researcher demonstrated the effects of wogonin on IL-1β-stimulated OA human chondrocytes and cartilage explants. Wogonin significantly inhibits the production, gene expression, and activity of inflammatory mediators, oxidative stress, and MMP including IL-6, iNOS, NO, COX-2, PGE_2_, ROS, MMP3, MMP9, MMP13, and ADAMTS4. Furthermore, it also inhibits the collagenase activity and reverses the suppression of collagen type II and aggrecan expressions induced by IL-1β.

In the cartilage explants, wogonin significantly blocks the IL-1β-induced release of collagen type II and sGAG depending on the dosage used and it is also able to prevent proteoglycan loss from the cartilage. From the results above, wogonin is proven to exhibit a protective ability toward chondrocytes and cartilage. Apart from that, wogonin can enhance the expression of Nrf-2-dependent antioxidants and various chondroprotective enzymes including SOD-2, NQO1, glutamate-cysteine ligase catalytic subunit (GCLC), and HO-1. This further confirms that wogonin exerts chondroprotective properties [90,123]. In the study by Park et al. (2015), wogonin successfully inhibits gene expression and the production of MMP1, MMP3, MMP13, and ADAMTS4. Chondrocytes treated with wogonin showed an increase in the expression of collagen type II. In an in vivo model, a decrease in the MMP3 production was observed in the OA rat model [85]. These studies suggest that wogonin may be a promising antioxidant candidate for OA treatment.

## 7. Catabolic and Anabolic Effect of Natural Compounds in OA

The usage of certain natural compounds for treating OA inhibits catabolic and anabolic factors. For instance, as tabulated in Table 1, edible bird’s nest [79] and genistein [91] from soybean are able to inhibit catabolic factors from 24 h up to 7 days in an in vitro testing. It mainly focuses on the inhibition of catabolic factors such as nitrite, iNOS, ROS, PGE2, COX-2, MMP1, MMP2, MMP3, MMP9, MMP13, ADAMTS4, IL-1, IL-6, and IL-8 while cynaroside [55] is able to inhibit both catabolic and anabolic factors including collage type II and aggrecan when being exposed to OA-induced chondrocytes. Inhibition of iNOS and nitrite prevents damage to cartilages by hindering the activity of MMP [35] while suppression of PGE2 restores homeostasis in the bone [125]. Similarly, the inhibition of COX-2 modulates controlled apoptosis and proliferation and reduces the release of PGE2 in the articular cartilage thus leading to healthy chondrocytes [126]. MMPs usually have a distinct role in the pathology of osteoarthritis and all types of MMPs have their specialized catalytic domain. They usually stay inactive in the presence of N-terminal pro-peptide. Upon activation, proteolytic from the pro-peptides will be removed from its cellular sites in accordance with the proteinase. All types of MMPs have their specific substrate cleavage that has a role in the progression of any condition [127]. For instance, inhibition of catabolic factors such as MMP1 and MMP13 or MMP 9 (inducible type of MMP) prevents the cleavage of the triple helix of collagen thereby preventing the damage to interstitial collagens in the arthritic joints [128]. Meanwhile, MMP2 has the potential to inhibit inflammatory factors or stimulate the anti-inflammatory factors that contribute to the progression of OA [129].

Furthermore, the function of ADAMTS4 is to mediate the degradation of aggrecan independently without the involvement of MMPs in the cartilages [130]. Therefore, the inhibition of ADAMTS4 by the natural compounds ensures a cartilage protective mechanism. In contrast, elevation in the pro-inflammatory cytokines such as IL-1, IL-6, and IL-8 is able to trigger degradation in the ECM, loss of collagen, as well as sulfated proteoglycans. Such alteration is closely associated with the inflammatory process for OA progression [131]. However, natural compounds such as edible bird’s nest [79] and genistein [91] have the ability to inhibit such catabolic factors, thus preventing the worsening of OA pathology. 

## 8. Conclusions

In conclusion, this review recapitulated the effect of natural compounds in treating OA pathology focusing on the oxidative stress signaling pathway. In short, there are various downstream mediators—such as MAPK, NF-κβ, PI3K/Akt, and JNK—that are involved in the oxidative stress signaling pathway of OA progression. There are various studies which suggest that the extracts or components from natural products exhibit chondroprotective properties. This study highlights the importance and the potential of natural products in becoming candidates as antioxidant agents for OA. The articles included in this review specifically concentrate on the therapeutic effect of selected natural compounds that inhibit catabolic and anabolic factors in OA. As such, this review could provide insight for future researchers to deepen their research on treating OA or in developing a new therapeutic drug based on the natural compounds focusing on oxidative stress signaling mechanism.

## Figures and Tables

**Table 1:** In vitro evidence of the effects of natural compound on osteoarthritis.

Author	Subject	Active Compound	Route of Treatment	Duration of Treatment	Dosage	Cell Lines/Types	Types	Biological Effects/Findings
Miller et al., 2007 [69]	*Croton palanostigma*	Progrado (proanthocy-anidin)	Cell seeding	72 h	2 and 10 μg/mL	IL-1β treated human chondrocytes	Monolayer culture	-Reduce glycosaminoglycan release, block cartilage catabolism -Block gelatinolytic activity of MMP2, MMP9 -Promotes production of cartilage repair factor, insulin-like growth factor 1 (IGF-1)
Shakibaei et al., 2007 [70]	Not stated	Resveratrol	Cell seeding	0, 1, 12, and 24 h	100 μM	IL-1β treated human articular chondrocytes	Monolayer culture	-Increased level of collagen type II and β1-integrin synthesize -Deactivation of caspase-3 and cleavage of PARP
Sumantran et al., 2007 [71]	*Withania somnifera* (root)	*Withania somnifera*	Cell seeding	2, 4, 6, and 8 days	0.05, 0.1, 0.5, or 1 mg/mL	Human explant cartilage	Monolayer culture	-Reduce proteoglycan release -Inhibit collagenase type II
Sumantran et al., 2008 [72]	*Phyllanthis emblicai* (fruit)	*Phyllanthis emblicai*	Cell seeding	2,4,6, and 8 days	0.05 or 0.10 mg/mL	Human explant cartilage	Monolayer culture	-Inhibit hyaluronidase and collagenase type II activity -Reduce sGAG release
Sanyacharernkul et al., 2009 [73]	*Dregea volubilis* Benth. ex Hook. f (roots)	Polyoxypregnane glycoside	Cell seeding	3 days	6.25, 12.5, and 25 µg mL^−1^	IL-1β treated porcine cartilage	Monolayer culture	-Reduce sGAG, HA, and MMP2 activity -Reverse effect of IL-1β-reduced UA level
Liu et al., 2010 [74]	Grape (skin)	Resveratrol	Cell seeding	24 h	25, 50, 75, and 100 μM	Advanced glycation end products (AGE)-stimulated OA porcine chondrocytes and cartilage explants	Monolayer culture	-Suppress iNOS-NO and COX-2-PGE2 production -Inhibit IKK-IκBα-NF-κβ and JNK/ERK-activator protein-1 (AP-1) signaling pathways -Decrease MMP13 and prevent destruction of collage type II -Prevent degradation of proteoglycan and aggrecan in explant cartilage
Hu et al., 2011 [75]	*Hydrastis canadensis* (goldenseal), *Cortex phellodendri* (Huang bai), and *Rhizoma coptidis* (Huang lian)	Barberine	Cell seeding	3 days	10, 25, 50, 100, µm	IL-1β treated rat articular chondrocytes and cartilage explants	Monolayer culture	-Reduce sGAG release -Inhibit NO production -Suppress production of MMP1, MMP3, and MMP13 -Upregulate TIMP-1 -Decrease lesions severity
Chai et al., 2012 [76]	*Zingiber cassumunar* Roxb.	cis-3-(2′,4′,5′-trimethoxyphenyl)-4-{(E)-2′′′,4′′′,5′′′-trimethoxystyryl}cyclohex-1-ene (Compound C) and (E)-4-(3′,4′-dimethoxyphenyl)but-3-en-1-ol (Compound D)	Cell seeding	3 days	1, 10, or 100 µM	IL-1β treated porcine cartilage explant	Monolayer culture	-Conserve collagen and UA -Reduce sGAG and HA release -Inhibit MMP2 and MMP13 activity
Itthiarbha et al., 2012 [77]	*Dregea volubilis*	Polyoxypregnane glycoside	Cell seeding	3 days	6.25, 12.5, and 25 µg mL^−1^	IL-1β treated human articular chondrocytes	Monolayer	-Decrease HA released -Inhibit expression of MMP1, MMP3, and MMP13 -Reverse effect of IL-1β-inhibited type II collagen expression -Inhibit IL-1β-induced HA and MMPs mRNA upregulation -Inhibit phosphorylation of IKK and IκBα degradation of NF-κβ signaling pathway
Phitak et al., 2012 [78]	*Sesamun indicum* Linn.	Sesamin	Cell seeding	24 h	0.25, 0.5, and 1.0 μM	IL-1β treated porcine cartilage explants	Monolayer culture	-Inhibit release of sGAG and hydroxyproline -Inhibit loss of UA, prevent proteoglycan degradation -Decrease expression and activity of MMP1, MMP3, and MMP13 -Inhibit p38 and JNK signaling pathway
Chua et al., 2013 [79]	Edible bird’s nest (swiftlets’ saliva)	Not stated	Cell seeding	7 days	0.05–3.00%	Human articular chondrocytes	Monolayer culture	-Promote human articular cartilage proliferation -Reduce catabolic genes’ expression (MMP1, MMP3, IL-1, IL-6, IL-8, COX-2, iNOS, PGE2) -Increase type II collagen, aggrecan, and transcription factor SOX-9 gene expression -Increase sGAG production
Liu et al., 2014 [80]	Resveratrol	Resveratrol	Cell seeding	24 h	6.25, 12.5, 25, 50, 100, or 200 μM	IL-1β treated human knee articular chondrocytes	Monolayer culture	-Decreased level of IL-1β and TNFα -Decreased expression of TLR4 mRNA levels -Downregulation of TLR4/NF-κβ signaling pathway -Decreased expression of MyD88 and TRAF6
Tong et al., 2014 [81]	*Aconitum carmichaelii* Debx. (Fuzi)	Not stated	Cell seeding	24, 48, and 72 h	5% to 20%	Mono-iodoacetate (MIA) treated rat chondrocytes	Monolayer culture	-Promote chondrocyte proliferation, inhibit chondrocyte damage
Rufino et al., 2014 [82]	*Juniperus oxycedrus* L. subsp. Oxycedrus (leaves)	α-pinene	Cell seeding	24 h	10, 25, 50, 100, 125, and 200 μg/mL	IL-1β treated human articular chondrocytes and human chondrocytes cell line (C28/12)	Monolayer culture	-Inhibit phosphorylation of IκBα, inhibit NF-κβ and JNK activation -Inhibit expression of iNOS, MMP1, and MMP13 genes
Pradit et al., 2014 [83]	*Phyllanthus amarus* Schum. and Thonn.	Phyllanthin and hypophyllanthin	Cell seeding	14 days	50, 100, and 200 μg/mL	IL-1β treated porcine articular cartilage explants	Monolayer culture	-Decrease sGAG level and MMP2 activity -Increase UA and proteoglycan contents
Wen et al., 2015 [84]	Gallnuts, grapes, tea leaves, and oak bark	Gallic acid	Cell seeding	48 h	0, 10, 20, 40, 80 μM	AGE-treated rabbit chondrocytes	Monolayer culture	-Inhibit ROS release -Suppress SOD and GSH activities -Reverse collagen type II and aggrecan degradation -Inhibit expression of iNOS-NO and COX-2-PGE2
Liu et al., 2015 [56]	*Rhizoma coptidis* (Huang Lian)	Berberine	Cell seeding	24 h	1, 3, 10, and 30 ng/mL	Connective tissue growth factor (CNN2)-induced IL-1β expression treated human synovial fibroblast	Monolayer culture	-Attenuate CCN2-induced IL-1β expression -Reduce ROS generation -Reduce phosphorylation of apoptosis signal-regulating kinase 1 (ASK1), p38, JNK, and p65
Park et al., 2015 [85]	*Scutellariae radix*	Wogonin	Cell seeding	24 h	1, 10, 50, or 100 μM	IL-1β treated rabbit articular chondrocytes	Monolayer culture	-Inhibit MMP1, MMP3, MMP13, and ADAMTS4 gene expression -Increase collagen type II expression -Inhibit MMP3 production and activity
Zhang et al., 2016 [63]	Turmeric	Curcumin	Cell seeding	6 h	100 μM	IL-1β treated human chondrocytes	Monolayer culture	-Suppress mRNA expression of IL-1β, TNF-α, MMP1, MMP3, MMP13, and ADAMTS5 -Upregulate chondroprotective transcriptional regulator, Cbp/p300 interacting transactivator with ED-rich tail 2 (CITED2) -Reduce OA disease progression
Buddhachat et al., 2016 [86]	*Phyllanthus amarus*, *Phyllanthus urinaria* L., *Phyllanthus urinaria* subsp. chamaepeuce, *Phyllanthus debilis*, and *Phyllanthus airy-shawii*	Phyllanthin and hypophyllanthin	Cell seeding	2 weeks	50, 100, and 250 µg/mL.	IL-1β treated porcine cartilage cartilage explants	Monolayer culture	-Inhibit sGAG release -Reduce loss of UA level -Reduce proteoglycan degradation
Kong et al., 2016 [87]	Sesame	Sesamin	Cell seeding	24 h	2.5, 5, or 10 μM	IL-1β treated human chondrocytes	Monolayer culture	-Inhibit PGE2 and NO production -Inhibit production of MMP1, MMP3, and MMP13 -Block NF-κβ p65 and IκBα phosphorylation -Increase expression of Nrf-2 and HO-1
Liu et al., 2017 [88]	Resveratrol	Resveratrol	Cell seeding	48 h	10 µM	IL-1β treated human chondrocytes	Pellet culture	-Increased expression of Sirtuin 1 (Sirt1) -Decreased level of cell apoptosis -Decreased expression of procaspase-3, -9, MMP1, MMP3, MMP13, Wnt3α, Wnt5 α, Wnt7 α, and β-catenin
Gu et al., 2017 [89]	Resveratrol	Resveratrol	Cell seeding	24 h	0, 6.25, 12.5, 25, 50, 100, and 200 µM	IL-1β treated human chondrocytes	Monolayer culture	-Decreased level of MMP-13 and IL-6 -Suppression of TLR4/MyD88 dependent and independent downstream pathways
Khan et al., 2017 [90]	*Scutellaria baicalensis* (root)	Wogonin	Cell seeding	2 h	10, 25, 50, or 100 µM	IL-1β-stimulated human OA chondrocytes and cartilage explants	Monolayer culture	-Inhibit expression of IL-6, iNOS-NO, and COX-2-PGE2 -Suppress ROS production -Reduce MMP3, MMP9, MMP13, and ADAMTS4 mRNA expression and protein production -Enhance collagen type II and aggrecan mRNA and protein expression -Inhibit release of collagen type II and sGAG by cartilage explants -Reduce proteoglycan release from cartilage explants -Increase expression of Nrf-2-dependent antioxidant and cytoprotective enzymes (SOD-2, NQO1, glutamate-cysteine ligase catalytic subunit (GCLC),HO-1)
Sun et al., 2019 [59]	Astaxanthin	Astaxanthin	Cell seeding	2 h	5, 10, and 20 μM	IL-1β, TNF-α, and tert-butyl hydroperoxide (TBHP) treated chondrocytes	Monolayer culture	-Reduce ADAMTS5, MMP3, and MMP13 mRNA level -Increase expression of collagen type II -Upregulation of nuclear factor (erythroid-derived 2)-like 2 (Nrf-2) signaling and downstream chondroprotective gene, heme oxygenase-1 (HO-1) and reduced nicotinamide adenine dinucleotide phosphate (NADPH) quinone oxidoreductase (NQO1) -Reduce iNOS and COX-2 protein level -Block phosphorylation of ERK and JNK, block MAPK signaling -Inhibit p65 and IκBα phosphorylation, inhibit NF-κβ signaling -Reduce ROS level
Liu et al., 2019 [91]	*Glycine max* (soybean)	Genistein	Cell seeding	24 h	5, 10, and 50 μM	IL-1β treated human articular chondrocytes	Monolayer culture	-Inhibit expression of catabolic factors (nitric oxide synthase 2 (NOS2), COX-2, MMP1, MMP2, MMP3, MMP9, and MMP13 -Stimulate HO-1expression in Nrf-2 pathway activation
Ongchai et al., 2019 [92]	*Senna alata* (L.) Roxb. and *Senna tora* (L.) Roxb. (leaves)	Rhein / aloe-emodin	Cell seeding	7, 14, and 21 days	1.25, 2.5, or 5 μg/mL	IL-17α and IL-1β treated porcine cartilage explants	Monolayer culture	-Prevent cartilage degradation -Reduce sGAG and HA release, preserve proteoglycan
Heikal et al., 2019 [93]	*Stichopus chloronotus* (sea cucumber)	Stichopus chloronotus	Cell seeding	7 days	0.1, 0.2, 0.3, 0.4, 0.5, 0.6, 0.7, 0.8, 0.9, and 1.0%	Human articular chondrocytes	Monolayer culture	-Maintain chondrocytes characteristics -Increase collagen type II, aggrecan, and SOX-9 gene expression -Reduce gene expression of collagen type I, IL-1, IL-6, IL-8, MMP1, MMP3, MMP13, COX-2, iNOS, and proteinase-activated receptor 2 (PAR-2) -Increase production of sGAG -Reduce production of PGE2 and NO
D’Ascola et al., 2019 [94]	Curcumin	Curcumin	Cell seeding	16 h	0.65, 1.25, 2.5, 5, and 10 µg/mL	Human articular chondrocytes	Monolayer culture	-Decreased expression of IL-1β -Decreased expression of NF-κβ and STAT3 -Increased expression of collagen type II
Zhang et al., 2020 [67]	*Aconitum carmichaelii* Debeaux derived Hei-shun-pian (Hsp)	Benzoylacon-itine and benzoylhypa-conitine	Cell seeding	24, 48, and 72 h	2.5, 5, 10, 15%	TNF-α treated rat articular chondrocytes	Monolayer culture	-Stimulate chondrocytes proliferation -Regulate collagen type II, MMP1, ADAMTS9, and aggrecan gene expression
Lee et al., 2020 [55]	Cynaroside	Cynaroside	Cell seeding	24 h	0, 40, 80, and 160μM	IL-1β treated chondrocytes	Monolayer culture	-Inhibit expression of catabolic factors (nitrite, iNOS, ROS, PGE2, COX-2, MMP1, MMP3, MMP13, ADAMTS4) -Inhibit degradation of anabolic factors (collage type II and aggrecan) -Suppress phosphorylation of MAPKS and translocation of NF-κβ p65 subunit -Inhibit production of nitrite and PGE2 -Suppress protein expression of iNOS, COX-2, MMP13, and ADAMTS4 -Inhibit proteoglycan degradation
Lee et al., 2020 [95]	*Artemisia argyi* (seomae mugwort)	Jaceosidin	Cell seeding	24 h	10, 50, and 100 μg/mL	In vitro—IL-1β, IL-6 and TNF-α treated chondrocytes	Monolayer culture	-Suppress MMP3, MMP13, ADAMTS4, and ADAMTS5 expression -Reduce aggrecanase activity -Protect against sulfated proteoglycan degradation
You et al., 2021 [96]	*Illicium verum*	Shikimic acid (SA)	Cell seeding	24 h	0.1, 1, 5, 10, and 20 mM	IL-1β treated human chondrocytes (SW1353)	Monolayer culture	-Decrease expression of iNOS, COX-2, MMP3, MMP13, ADAMTS5, collagen type X and p63 -Increase collagen type II, autophagy related 7 (ATG7), beclin-1, and microtubule-associated light chain-3 (LC3) expression -Increase autophagic flux -Suppress activation of MAPK and NF-κβ pathway
Buhrmann et al., 2020 [97]	Curcumin	Curcumin	Cell seeding	10 days	1, 2, 5, and 10 μM	3D-chondrocyte-alginate beads	Monolayer culture	-Suppress activation of NF-κβ pathway -Increased expression of collagen type II-, cartilage-specific proteoglycans, β1-integrin, and Sox9 -Downregulation of MMP-9, cox-2, and caspase-3
Xu et al., 2022 [98]	Curcumin	Curcumin	Cell seeding	2 days	10 µM/L	Tert-butyl hydroperoxide (TBHP) treated C57 mice chondrocytes	Monolayer culture	-Increased chondrocytes proliferation -Increased extracellular matrix anabolism -Decreased level of (aggrecan, collagen II, ADAMTS5, MMP13, IL-1β, and TNFα -Decreased level of 8-OHdG positive cells and MDA

**Table 2:** In vivo evidence of the effects of natural compounds on osteoarthritis.

Author	Subject	Active Compound	Route of Treatment	Duration of Treatment	Dosage	Model Type	Anatomical Location	Biological Effects / Findings
El-Ghazaly et al., 2011 [52]	Propolis	Propolis	Intraarticular injection	Once daily for 2 weeks	5 mL/kg	Male wistar rats injected with Freud’s adjuvant injected	Right hind paw	-Reduce breakdown of cartilage -Prevent reduction sulfated glycosaminoglycan (sGAG) and hydroxyproline level -Reduce TNF-α level -Normalize NO and the oxidative stress biomarkers (GSH, MDA)
Hu et al., 2011 [75]	*Hydrastis canadensis* (goldenseal), *Cortex phellodendri* (Huang bai), and *Rhizoma coptidis* (Huang lian)	Barberine	Intraarticular injection	Not specified	10, 50, or 100 µm	Male SD rats injected with IL-1β	Right knee joint	-Reduce MMP1, MMP3, and MMP13 gene expression and protein production -Increase TIMP-1 mRNA levels and protein expression
Wang et al., 2011 [99]	Resveratrol	Resveratrol	Intraarticular injection	2 weeks	50, 20, and 10 µmol/kg	1.5 to 2.5 kg male and female rabbits subjected to medial parapatellar arthrotomy	Right knee	-Decreased level of proteoglycan loss -Decreased level of cell apoptosis in chondrocytes -Decreased level of nitric oxide
Phitak et al., 2012 [78]	*Sesamun indicum* Linn.	Sesamin	Intraarticular injection	5 days for 5 weeks	1 μM or 10 μM	8-week-old Wistar rats injected with papain	Right knee	-Reverse pathological changes -Reduce chondrocyte disorganization -Increase cartilage thickness -Decrease collagen type II and proteoglycan loss -Increase formation of collagen type II and proteoglycan
Leong et al., 2014 [64]	Green tea	Epigallocatechin 3-gallate (EGCG)	Intraperitoneal injection	4 weeks	25 mg/kg	5- to 6-month-old male SD rats surgically transecting the medial meniscotibial ligament (MMTL)	Destabilization of the medial meniscus (DMM) at the right hind limb	-Slows progression of early and midstage OA -Reduce degradation of aggrecan and collagen type II -Reduce MMP13 and ADAMTS5 -Reduce MMP1, MMP3, MMP8, MMP13, ADAMTS5, IL-1β, and TNF-α mRNA levels -Elevate gene expression of CITED2 -Reduce expression of IL-1β, TNF-α, and chemokine receptor Ccr2 in dorsal root ganglion (DRG)
Hanprasertpong et al., 2014 [66]	*Cryptolepis buchanani* Roem. and Schult.	*Cryptolepis buchanani*	Intraperitoneal injection	5 h	100, 250, and 500 mg/kg	Male SD rats and Swiss albino mice injected with carrageenan	Hind paw	-Reduced MMP-2 activity
Tong et al., 2014 [81]	*Aconitum carmichaelii* Debx (Fuzi)	Not stated	Oral administration	28 days	Daily 14 g/kg	Male rats injected with MIA	Right knee joint	-Prevent alteration in joint morphology (osteolysis, swelling, and irregular catilage surface) and bone density -Prevent subchondral abonormalities (loss of cartilage, cyst, fibrillation of bone marrow, subchondral sclerosis, and disorganized chondrocutes culture) -Show chondroprotective activity
Jeong et al., 2015 [68]	*Artemsia princeps* Pampanini	Eupatilin	Oral administration	14 days	100 mg/kg	6-week-old male Wistar rats injected with MIA	Patellar ligament of right knee	-Reduce osteoclasts number -Reduce expression of IL-1β, IL-6, nitrotyrosine, iNOS -Reduce mRNA of MMP3, MMP13, and ADAMTS5 by increasing tissue inhibitor of metalloproteinases-1 (TIMP-1) -Inhibit phosphorylation of JNK protein -Suppress oxidative damage, enhance ECM production
Wen et al., 2015 [84]	Gallnuts, grapes, tea leaves, and oak bark	Gallic acid	Intra-articular injection	Once every 72 h for 8 weeks	80 μM	Rabbits injected with collagenase type II	Right knee	-Reduce cartilage degradation
Liu et al., 2015 [56]	*Rhizoma coptidis* (Huang Lian)	Berberine	Intraperitoneal injections	30 days	10 or 30 mg/kg	Rats injected with collagenase	Right knee joint	-Inhibit OA development -Lower IL-1β serum level -Reduce proteoglycan break down
Park et al., 2015 [85]	*Scutellariae radix*	Wogonin	Intraarticular injection	7 h	50 or 100 μM	Male SD rats injected with IL-1β	Right knee joint	-Inhibit production of MMP3
Zhang et al., 2016 [63]	Turmeric	Curcumin	Oral administration	8 weeks	50 mg/kg	5- to 6-week-old male mice subjected to MMTL surgery	DMM at the right hind limb	-Reduce proteoglycan loss and cartilage erosion -Reduce synovitis and subchondral plate thickness -Reduce type II collagen and aggrecan cleavage and MMP13 and ADAMTS positive chondrocytes
Gu et al., 2016 [100]	Resveratrol	Resveratrol	Oral administration	12 weeks	5, 22.5, or 45 mg/kg	7-week-old male C57BL/6J mice high fat diet (HFD) induced OA	Not specified	-Improvement in arrangement of matrix, and tide lines in chondrocytes -Decreased level of CTX-II -Increased expression of type II collagen -Decreased level of TUNEL-positive cells
Wei et al., 2017 [101]	Resveratrol	Resveratrol	Oral administration	8 weeks	50 mg/kg	6-week-old male Wistar rats subjected to MIA injection	Patellar ligament of right knee	-Decreased expression of TNF-α, IL-1β, IL-6, and IL-18 -Suppression of iNOS, NF-κβ, p-AMPK, and SIRT1 protein -Increased level of SOD -Decreased level of MDA -Increased leve of HO-1 and Nrf-2 -Decreased activity of caspase-3/9
Zhang et al., 2017 [102]	Curcumin	Curcumin	Oral administration	8 weeks	50 mg/kg	8-week-old male C57BL/6 mice subjected to MMTL surgery	DMM at right knee	-Decreased cartilage loss and chondrocytes apoptotis -Suppression of caspase-3 expression -Decreased expression of LC-I/II and beclin 1 -Decreased expression of mTOR, Akt, and p-P70S6k
Jiang et al., 2017 [103]	Resveratrol	Resveratrol	Oral administration	12 weeks	22.5 or 50 mg/kg	7-week-old male C57BL/6J mice HFD induced OA	Not specified	-Increased cartilage thickness and improvement on chrondrocytes arrangements -Decrease expression of TLR4 and tumor necrosis factor receptor-associated factor 6 (TRAF6)
Sun et al., 2017 [104]	Curcumin	Curcumin	Intraperitoneal injection	8 weeks	50 mM	Male C57BL/6 mice subjected to MMTL surgery	DMM	-Decreased level of IL-1b, IFN-g, IL-17A, IL-18, TNF-a, and VCAM1 -Increased interaction of pro-caspase-1 to NLRP3 -Decreased level of caspase-1 activation
Lee et al., 2018 [54]	*Anthriscus sylvestris* (leaves)	Anthriscus sylvestris	Oral administration	8 weeks	50, 100, and 200 mg/kg body	8-week-old male rats subjected to MMTL surgery	DMM at right knee	-Inhibit cartilage destruction and proteolycan loss
Liu et al., 2019 [91]	*Glycine max* (soybean)	Genistein	Oral administration	12 weeks	40 mg/kg	8-weeks-old male SD rats subjected to anterior cruciate ligament transection (ACLT)	Not specified	-Slow down progression of OA
Yan et al., 2019 [105]	Curcumin	Curcumin	Intra-articular injection	8 weeks	50 μl	Male SD rats subjected to ACLT	Right knee	-Suppression of TLR4 activation -Decreased level of IL-1β and TNFα -Suppression of NF-κβ
Feng et al., 2019 [106]	Curcumin	Curcumin	Intraperitoneal injection	8 weeks	50 and 150 mg/kg	8-week-old male SD rats subjected ACLT	Right knee	-Downregulation of caspase3 and PARP protein -Increased expression of SIRT1 -Reverse the loss of chondrocytes and proteoglycan
Jiang et al., 2020 [107]	Curcumin	Curcumin	Intra-articular injection	4 weeks	40 μM	8-week-old male SD rats injected with Freud’s adjuvant	Temporomandibular joint osteoarthritis	-Decreased expression of mRNA levels of COL2A1 and ACAN -Increased level of Nrf2 and p-Nrf2 -Decreased level of proteoglycan loss -Decreased level of iNOS, COX-2, IL-1β, MMP-9, and MMP-13
Susmiarsih et al., 2019 [108]	Green Tea	EGCG	Oral administration	8 weeks	200 mg/kg	Rabbits injected with Freud’s adjuvant	Not specified	-Decrease degree of knee joint damage -Decrease NO level
Sun et al., 2019 [59]	Astaxanthin	Astaxanthin	Intra-articular injection	2 times a week for 8 weeks	20 mg/kg	8-week-old male C57BL/6 mice subjected to MMTL to surgery	DMM at the right knee	-Reduce cartilage degradation
Lee et al., 2020 [95]	*Artemisia argyi* (seomae mugwort)	Jaceosidin	Oral administration	10 weeks	50, 100, and 250 mg/kg	10-week-old male C57BL/6 mice subjected to MMTL surgery	DMM	-Suppress cartilage destruction and reduce subchondral bone thickness
Zhang et al., 2020 [67]	*Aconitum carmichaelii* Debeaux derived Hei-shun-pian (Hsp)	Benzoylacon-itine and benzoylhypa-conitine	Oral administration	28 days	14 g/kg dHsp	Male and female rats injceted with MIA	Patellar ligament of rat knee	-Prevent articular degradation -Restore abnormal expression of collagen type X, MMP2, SOX-5, ADAMTS4, ADAMTS5 and ADAMTS9 -Increase collagen type II expression
Huang et al., 2021 [50]	Propolis	Galangin	Intra-articular injection	8 weeks	5 mg/ml	8-week-old male rats subjected to ACLT	Right knee	-Reduce cartilage degradation
Su et al., 2021 [51]	Galangin	Galangin	Oral administration	14 days	10 and 100 mg/kg	Male SD rats induced with MIA	Left femorotibial joint	-Reduce urinary type II collagen -Reduce reactive oxygen species (ROS), lipid peroxidation, IL-1β, IL-6, and TNF-α -Increase catalase, superoxide dismutase (SOD), glutathione peroxidase (Gpx), and reduce glutathione (GSH) level -Reduce collagen type II mRNA and protein expression level indicate inhibit cartilage degradation
You et al., 2021 [96]	*Illicium verum*	Shikimic acid (SA)	Intra-articular injection	2 times a week for 1 month	20 mM in 100 µL	6-week-old SD male rats subjected to ACLT	Right knee	-Mitigates cartilage degradation -Alleviate OA progression
Xu et al., 2022 [98]	Curcumin	Curcumin	Intra-articular injection	Biweekly for 4 weeks	1 × 10^9^ p/mL	Male C57 mice subjected to ACLT	Right knee	-Alleviate cartiage degeneration and abrasion -Decreased level of aggrecan and collagen II -Decreased level of cleaved caspase 3 and 8-OHdG
